# Association Between Use of Anti-gout Preparations and Dementia: Nested Case–Control Nationwide Population-Based Cohort Study

**DOI:** 10.3389/fmed.2020.607808

**Published:** 2021-01-12

**Authors:** Tsung-Ju Chuang, Yu-Hsun Wang, James Cheng-Chung Wei, Chih-Jung Yeh

**Affiliations:** ^1^Division of Endocrinology and Metabolism, Department of Internal Medicine, National Defense Medical Center, Taichung Armed Forces General Hospital, Taichung, Taiwan; ^2^School of Public Health, Chung Shan Medical University, Taichung, Taiwan; ^3^Department of Medical Research, Chung Shan Medical University Hospital, Taichung, Taiwan; ^4^Department of Allergy, Immunology and Rheumatology, Chung Shan Medical University Hospital, Taichung, Taiwan; ^5^Institute of Medicine, College of Medicine, Chung Shan Medical University, Taichung, Taiwan; ^6^Graduate Institute of Integrated Medicine, China Medical University, Taichung, Taiwan

**Keywords:** gout, uric acid, dementia, benzbromarone, elderly

## Abstract

**Objectives:** Gout is the most common form of inflammatory arthritis and was found to be independently associated with incident dementia in the elderly. However, the associations between anti-gout preparations and dementia were not well-studied.

**Methods:** Data were collected from Taiwan's National Health Insurance Research Database (NHIRD). A 2005–2013 retrospective cohort study was conducted, and all investigated subjects were identified by International Statistical Classification of Diseases and Related Health Problems, 9th Revision, Clinical Modification. Conditional logistic regression was used to evaluate the odds ratio of dementia in relation to different gout preparations (benzbromarone, allopurinol, sulfinpyrazone, probenecid) and number of days of anti-gout preparation use, after adjustment for potential confounding variables.

**Results:** A total of 3,242 gout patients with and without dementia were selected from the NHIRD and included in the final analysis after 1:1 matching for age, gender, and diagnosis year of gout. In the anti-gout preparations, only use of Benzbromarone decreased the risk of dementia (adjusted OR, 0.81; 95% CI, 0.68–0.97). The result of the subgroup analysis revealed a trend toward a lower risk of dementia with longer use of benzbromarone. Use of benzbromarone for ≥180 days showed a significantly lower risk of dementia (adjusted OR, 0.72; 95% CI, 0.58–0.89). Moreover, the protective effect was more pronounced in males compared with females.

**Conclusion:** This cohort study reveals that gout patients taking benzbromarone are at a decreased risk of developing incident dementia, especially with longer use and in male. Further prospective trials are warranted to confirm our findings.

## Introduction

Gout is the most common form of inflammatory arthritis that is caused by the deposition of monosodium urate crystals in and around the joints (1). It is characterized by hyperuricemia, with serum or plasma urate concentrations >6.8 mg/dL, which is the approximate limit of urate solubility (2). Gout may present with different disease manifestations, such as recurrent flares of inflammatory arthritis, chronic arthropathy, accumulation of urate crystals in the form of tophaceous deposits, and uric acid (UA) nephrolithiasis (3). In addition to acute inflammation and chronic UA stone formation, gout has also been associated with various comorbidities, such as diabetes, hypertension, chronic kidney disease, and cardiovascular disease (4). In a recent study, gout was found to be independently associated with a 17–20% higher risk of incident dementia in the elderly (5). The prevalence of gout is estimated to range from 2.5% in Europe to 3.9% in the United States (6, 7).

Dementia is an important public health problem that can be caused by Alzheimer's disease (60–70%), vascular dementia (20%), and other conditions such as Parkinson's disease (8). Moreover, dementia is associated with limited functional ability and decreased quality of life, which can lead to loss of independence and increased morbidity and mortality (9–11). Hence, since gout is associated with an increased risk of dementia, prevention, and treatment are important.

Treatment for gout includes pharmacological urate-lowering therapy (ULT) to prevent gout flare, tophi formation, and related comorbidities (12). Several classes of anti-inflammatory agents are effective for the treatment of gout flares, including systemic and intra-articular glucocorticoids, non-steroidal anti-inflammatory drugs, colchicine, and biological agents that inhibit the action of interleukin-1β (3, 13). Following an acute flare, ULT usually includes xanthine oxidase inhibitors and uricosuric drugs, to achieve serum urate levels <6 mg/dL (<357 μmol/L), which is substantially below the urate solubility limit (12, 14, 15).

The drugs should be selected according to the cause of disease, comorbidities, and hepatic and renal functions. Xanthine oxidase inhibitor decreases the synthesis of uric acid by inhibiting the activity of xanthine oxidase. Commonly used drugs include allopurinol and febuxostat (2). Allopurinol, in adults, the starting dose is 50–100 mg/d, and the dose can be increased by 50–100 mg increments to a maximum dose of 600 mg/d in patients *until the* uric acid target is reached. The recommended dose is 50–100 mg/d for chronic kidney disease (CKD), stage 3 to 4, and is contraindicated in patients with CKD, stage 5 (16). Febuxostat is a selective inhibitor of xanthine oxidase. The initial dose is 20–40 mg/d, which can be titrated gradually to a maximum of 80 mg/d if the serum uric acid target is not reached. Febuxostat has better safety in patients with renal insufficiency because it is cleared mainly via the liver (17).

The primary uricosuric drugs include probenecid, benzbromarone, and sulfinpyrazone. Benzbromarone increases urinary excretion of uric acid and the starting dose is 25–50 mg/d in adults, which should be adjusted to 75 or 100 mg/d according to the serum UA level (18). Benzbromarone can be used in patients with mild-to-moderate renal dysfunction. The recommended dose is 50 mg/d in patients with an eGFR of 20–60 ml·min^−1^·1.73 m^−2^ and is contraindicated in patients with an eGFR <20 ml·min^−1^·1.73 m^−2^ or uric acid nephrolithiasis. The most concern adverse reaction of benzbromarone is hepatic toxicity (19). Probenecid can be started by 250 mg twice daily for 1 week and may increase to a maximum of 2 g/day. Moreover, Probenecid is avoided use in patients with eGFR <30 mL/min (20, 21). Sulfinpyrazone is started at a dose of 50 mg twice daily, with increments over several weeks to 100 to 200 mg three or four times daily as needed. The maximum effective dose of sulfinpyrazone is 800 mg/day, and avoid be used in persons with CKD or a history of uric acid kidney stones (22).

Like the description above, gout may be associated with various comorbidities including dementia; however, it is unclear whether anti-gout preparations can reduce the incidence of dementia. Hence, in the present study, we enrolled gout patients (newly diagnosed from 2000 to 2008) from the Taiwan Nation Health Insurance Research Database (NHIRD) to investigate the correlations of ULT and further dementia after at least 5 years from gout diagnosis.

## Methods

### Data Source

In 1995, Taiwan developed a health insurance system based on a single-payer government-run National Health Insurance (NHI) program, which currently includes comprehensive health care data for almost all Taiwanese citizens. The large, computerized databases that became the NHIRD were derived from this system by the Bureau of National Health Insurance and were maintained by the National Health Research Institutes, and include includes information about hospitalization, emergency care, and medical visits. These databases were used for research purposes, with a coverage rate >99% in 2010, and thus contains data for ~23 million beneficiaries (23).

The Longitudinal Health Insurance Research Dataset 2000 contains the clinical information of one million beneficiaries randomly selected from the NHIRD during the period from 2000 to 2013 (24). Moreover, the International Statistical Classification of Diseases and Related Health Problems, 9th Revision, Clinical Modification (ICD-9-CM) is used for the diagnosis by physicians. Patients' medical data include drug items, NHI code, dosage, frequency of use, and number of days prescribed. This information can be used to detecting drug interactions and potential duplicate medications when patients visit multiple hospitals. The NHI code corresponds to a code in the five-level Anatomical Therapeutic Chemical classification system recommended by the World Health Organization for studies on drug utilization (25).

### Data Collection

We identified one million people from the database. We selected patients aged ≥50 years with newly diagnosed gout based on the ICD-9-CM code 274 and use of anti-gout preparations, allopurinol (NHI code: M04AA01), benzbromarone (NHI code: M04AB03), sulfinpyrazone (NHI code: M04AB02), or probenecid (NHI code: M04AB01), within 6 months from 2000 to 2008. Dementia was defined as a diagnosis with one or more of the following ICD-9-CM codes: 290.0–290.4, 294.1, and 331.0–331.2. Moreover, the diagnosis of dementia before the first gout diagnosis date was excluded.

Individuals were divided into two groups. The dementia group comprised individuals newly diagnosed with dementia at following ≥2 outpatient assessments or a single inpatient admission after diagnosis of gout and 5 years apart. The non-dementia (control) group comprised of individuals who had never been diagnosed with dementia after being diagnosed with gout. The index date was set at 5 years after taking anti-gout preparations. The ratio of patients in dementia to non-dementia groups was 1:1, with individual matching for age, gender, and gout diagnosis year.

### Patient Subgroups

The age of the patients was defined on the index date. For the sex variable, zero represented female and one represented male. Baseline disease was diagnosed from two outpatient or one inpatient assessment 5 years before dementia diagnosis and included hypertension (ICD-9-CM = 401–405), hyperlipidemia (ICD-9-CM = 272.0–272.4), chronic liver disease (CLD; ICD-9-CM = 571), chronic kidney disease (CKD; ICD-9-CM = 585), diabetes (DM) (ICD-9-CM = 250), chronic obstructive pulmonary disease (COPD; ICD-9-CM = 491, 492, and 496), autoimmune disease (AD; ICD-9-CM =710, 714, and 720), cardiovascular disease (CVD; ICD-9-CM = 410–414), stroke (ICD-9-CM = 430–438), depression (ICD-9-CM = 296.2–3, 300.4, 311), and Parkinson's disease (PD; ICD-9-CM = 332). Patients with no comorbidities were represented by 0, and those who developed comorbidity were represented by 1. Moreover, use of warfarin (NHI code: B01AA03) or statins (NHI codes: C10AA01–05, 07–08) 5 years before dementia diagnosis were also included in the baseline characteristics.

### Statistical Analysis

All analyses were performed using SAS version 9.1.3 for Windows (SAS Institute, Inc., Cary, NC). *P*-values < 0.05 were considered statistically significant.

Kaplan–Meier analysis was applied to evaluate the cumulative incidence of dementia in the subgroups and log-rank test was to test for significance. Conditional logistic regression was used to evaluate the odds ratio (OR) of dementia in relation to different gout preparations, and number of days of anti-gout preparation use, after adjustment for potential confounding variables (allopurinol, benzbromarone, sulfinpyrazone, probenecid, hypertension, hyperlipidemia, CLD, CKD, DM, COPD, AD, CVD, stroke, warfarin, and statin). Subgroup analyses were performed by age (50–64 and ≥65 years) and gender.

## Results

**Figure 1:**
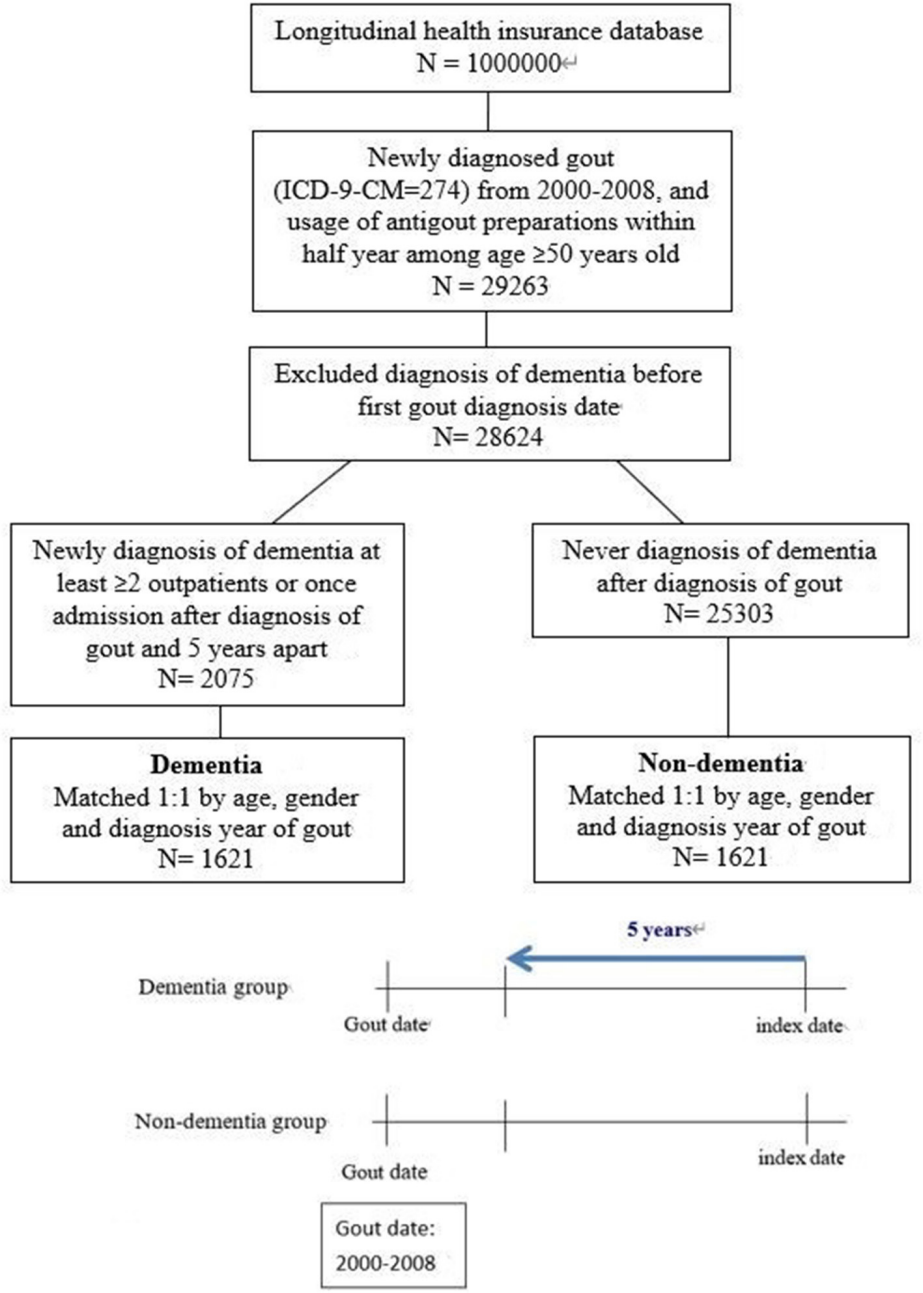
The study population selection protocol. Antigout preparations: use any one of Allopurinol, Benzbromarone, Sulfinpyrazone, and Probenecid.

A total of 3,242 gout patients with and without dementia were selected from the NHIRD and included in the final analysis after 1:1 matching for age, gender, and diagnosis year of gout. A flowchart of the study population selection protocol is shown in Figure 1.

Table 1 shows the demographic characteristics of the dementia and non-dementia group. The dementia group had higher prevalence of underlying disease include hypertension, DM, COPD, CVD, stroke, depression, and PD. Moreover, the dementia group had a higher prevalence of warfarin use and a similar prevalence of statin use compared with the non-dementia group. The most common anti-gout preparation used was benzbromarone (73.7% in the dementia group, 77.5% in the non-dementia group), followed by allopurinol (29.4% in the dementia group, 27% in the non-dementia group), sulfinpyrazone, and probenecid. Use of benzbromarone was significantly higher in the non-dementia group.

**Table 1:** Demographic characteristics.

	**Dementia (*****N*** **=** **1,621)**		**Non-dementia (*****N*** **=** **1,621)**		
	***n***	**%**	***n***	**%**	***p*-value**
Age					1
50–64	104	6.4	104	6.4	
65–79	920	56.8	920	56.8	
≥80	597	36.8	597	36.8	
Mean ± SD	76.9 ± 7.1		76.9 ± 7.1		1
Gender					1
Female	651	40.2	651	40.2	
Male	970	59.8	970	59.8	
Hypertension	1375	84.8	1272	78.5	<0.001
Hyperlipidemia	689	42.5	672	41.5	0.545
Chronic liver disease	332	20.5	302	18.6	0.184
Chronic kidney disease	223	13.8	171	10.5	0.005
Diabetes	638	39.4	538	33.2	<0.001
COPD	516	31.8	411	25.4	<0.001
Autoimmune disease	62	3.8	50	3.1	0.248
Cardiovascular disease	670	41.3	601	37.1	0.013
Stroke	760	46.9	357	22.0	<0.001
Depression	285	17.6	75	4.6	<0.001
Parkinson's disease	195	12.0	51	3.1	<0.001
Allopurinol	476	29.4	438	27.0	0.138
Benzbromarone	1,195	73.7	1,256	77.5	0.013
Sulfinpyrazone	72	4.4	64	3.9	0.483
Probenecid	18	1.1	10	0.6	0.129
Warfarin	96	5.9	65	4.0	0.012
Statin	631	38.9	591	36.5	0.147
Gout year					1
2000	539	33.3	539	33.3	
2001	328	20.2	328	20.2	
2002	247	15.2	247	15.2	
2003	178	11.0	178	11.0	
2004	124	7.6	124	7.6	
2005	90	5.6	90	5.6	
2006	61	3.8	61	3.8	
2007	36	2.2	36	2.2	
2008	18	1.1	18	1.1	
Study period (years)	7.8 ± 2.4		7.8 ± 2.4		0.917

*COPD, Chronic obstructive pulmonary disease*.

Table 2 shows the conditional logistic regression of risk of dementia. After adjusting for confunding factors, patients with underlying DM (OR, 1.21; 95% CI, 1.03–1.43), COPD (OR, 1.31; 95% CI, 1.10–1.55), and stroke (OR, 2.76; (95% CI, 2.35–3.24) showed an increased risk of dementia. Moreover, use of benzbromarone decreased the risk of dementia (adjusted OR, 0.81; 95% CI, 0.68–0.97).

**Table 2:** Conditional logistic regression of risk of dementia.

	**Crude OR**	**95% C.I**.	***p*-value**	**Adjusted OR^†^**	**95% C.I**.	***p*-value**
Allopurinol	1.13	0.97–1.32	0.130	1.02	0.86–1.22	0.809
Benzbromarone	0.82	0.70–0.96	0.013	0.81	0.68–0.97	0.021
Sulfinpyrazone	1.13	0.80–1.59	0.487	1.27	0.88–1.85	0.205
Probenecid	1.80	0.83–3.90	0.136	1.60	0.69–3.7	0.273
Hypertension	1.56	1.29–1.87	<0.001	1.20	0.97–1.47	0.087
Hyperlipidemia	1.04	0.91–1.20	0.543	0.96	0.80–1.16	0.704
Chronic liver disease	1.13	0.95–1.34	0.183	1.05	0.87–1.28	0.591
Chronic kidney disease	1.36	1.10–1.68	0.005	1.20	0.94–1.51	0.137
Diabetes	1.31	1.13–1.52	<0.001	1.21	1.03–1.43	0.021
COPD	1.40	1.20–1.65	<0.001	1.31	1.10–1.55	0.003
Autoimmune disease	1.26	0.86–1.84	0.245	1.27	0.84–1.92	0.257
Cardiovascular disease	1.19	1.04–1.37	0.014	1.01	0.86–1.19	0.879
Stroke	2.91	2.49–3.40	<0.001	2.76	2.35–3.24	<0.001
Depression	4.44	3.37–5.87	<0.001	3.45	2.57–4.63	<0.001
Parkinson's disease	4.20	3.03–5.81	<0.001	2.88	2.02–4.10	<0.001
Warfarin	1.53	1.10–2.12	0.012	1.17	0.82–1.67	0.388
Statin	1.11	0.96–1.29	0.141	0.94	0.77–1.14	0.515

*COPD, Chronic obstructive pulmonary disease*.

† *Adjusted for allopurinol, benzbromarone, sulfinpyrazone, probenecid, hypertension, hyperlipidemia, chronic liver disease, chronic kidney disease, diabetes, COPD, autoimmune disease, cardiovascular disease, stroke, depression, parkinson's disease, warfarin, and statin*.

The result of the subgroup analysis of anti-gout preparations divided by the number of days of use revealed a trend toward a lower risk of dementia with longer use of benzbromarone. Use of benzbromarone for ≥180 days showed a significantly lower risk of dementia (adjusted OR, 0.72; 95% CI, 0.58–0.89) (Table 3). Moreover, the protective effect of benzbromarone on dementia differed among the different age groups (Table 4). In adults aged 50–64 years, benzbromarone use lowered the risk of dementia with use for <90 days (adjusted OR, 0.21; 95% CI, 0.06–0.76), and use for ≥180 days (adjusted OR, 0.21; 95% CI, 0.06–0.76). In the elderly (≥65 years), benzbromarone use for ≥180 days decreased the risk of dementia (adjusted OR, 0.73; 95% CI, 0.59–0.92). Table 5 shows the different odd ratios in males and females. Both males and females showed a trend toward a decreased risk of dementia with longer use of benzbromarone. However, only use for ≥180 days showed a significant OR. Moreover, the protective effect was more pronounced in males (adjusted OR, 0.68) compared with females (adjusted OR, 0.72).

**Table 3:** Conditional logistic regression of risk of dementia divided by anti-gout preparations use days.

	***N***	**No. of dementia**	**Crude OR**	**95% C.I**.	***p*-value**	**Adjusted OR^†^**	**95% C.I**.	***p*-value**
Allopurinol (days)								
None	2,328	1,145	1			1		
<90	383	191	1.03	0.83–1.28	0.790	0.95	0.74–1.21	0.663
90–179	118	68	1.40	0.97–2.03	0.076	1.48	0.97–2.25	0.067
≥180	413	217	1.15	0.93–1.42	0.200	1.06	0.82–1.36	0.659
Benzbromarone (days)								
None	791	426	1			1		
<90	959	487	0.89	0.74–1.07	0.215	0.95	0.77–1.17	0.602
90–179	392	200	0.89	0.70–1.14	0.368	0.85	0.64–1.12	0.238
≥180	1,100	508	0.73	0.61–0.88	0.001	0.72	0.58–0.89	0.002
Sulfinpyrazone (days)								
None	3,106	1,549	1			1		
<90	57	30	1.11	0.66–1.87	0.691	1.34	0.74–2.43	0.335
≥90	79	42	1.14	0.73–1.80	0.564	1.41	0.84–2.35	0.194
Probenecid								
None	3,214	1,603	1			1		
Yes	28	18	1.80	0.83–3.90	0.136	1.68	0.73–3.89	0.224

† *Adjusted for allopurinol, benzbromarone, sulfinpyrazone, probenecid, hypertension, hyperlipidemia, chronic liver disease, chronic kidney disease, diabetes, COPD, autoimmune disease, cardiovascular disease, stroke, depression, parkinson's disease, warfarin, and statin*.

**Table 4:** Conditional logistic regression of risk of dementia divided by anti-gout preparations use days in different age group.

	***N***	**No. of dementia**	**Crude OR**	**95% C.I**.	***p*-value**	**Adjusted OR^†^**	**95% C.I**.	***p*-value**
**Age** **=** **50–64 (years)** Allopurinol (days)								
None	160	76	1			1		
<90	23	14	1.76	0.69–4.5	0.235	4.51	1.05–19.4	0.043
90–179	10	5	1.05	0.3–3.67	0.936	5.15	0.47–56.74	0.181
≥180	15	9	1.68	0.54–5.19	0.367	1.72	0.28–10.41	0.557
Benzbromarone (days)								
None	51	32	1			1		
<90	59	27	0.51	0.23–1.13	0.099	0.21	0.06–0.76	0.018
90–179	27	14	0.62	0.24–1.59	0.318	0.90	0.24–3.33	0.878
≥180	71	31	0.45	0.21–0.98	0.045	0.23	0.07–0.79	0.019
Sulfinpyrazone								
None	200	100	1			1		
Yes	8	4	1.00	0.25–4.00	1.000	0.48	0.07–3.48	0.469
Probenecid								
None	207	104	1			1		
Yes	1	0	NA	NA	NA	NA	NA	NA
**Age** **≥65 (years)** Allopurinol (days)								
None	2,168	1,069	1			1		
<90	360	177	1.00	0.79–1.25	0.977	0.91	0.71–1.18	0.475
90–179	108	63	1.43	0.97–2.11	0.070	1.41	0.91–2.18	0.122
≥180	398	208	1.13	0.91–1.40	0.274	1.03	0.80–1.34	0.796
Benzbromarone (days)								
None	740	394	1			1		
<90	900	460	0.92	0.76–1.12	0.400	0.98	0.79–1.22	0.850
90–179	365	186	0.92	0.71–1.18	0.496	0.86	0.64–1.15	0.300
≥180	1,029	477	0.75	0.62–0.91	0.004	0.73	0.59–0.92	0.006
Sulfinpyrazone (days)								
None	2,906	1,449	1			1		
<90	53	26	0.96	0.56–1.65	0.891	1.13	0.61–2.09	0.708
≥90	75	42	1.29	0.81–2.06	0.287	1.59	0.94–2.71	0.086
Probenecid								
None	3,007	1,499	1			1		
Yes	27	18	2.00	0.90–4.45	0.090	1.84	0.78–4.37	0.165

† *Adjusted for allopurinol, benzbromarone, sulfinpyrazone, probenecid, hypertension, hyperlipidemia, chronic liver disease, chronic kidney disease, diabetes, COPD, autoimmune disease, cardiovascular disease, stroke, depression, parkinson's disease, warfarin, and statin*.

**Table 5:** Conditional logistic regression of risk of dementia divided by anti-gout preparations use days in different sex.

	***N***	**No. of dementia**	**Crude OR**	**95% C.I**.	***p*-value**	**Adjusted OR^†^**	**95% C.I**.	***p*-value**
**Female** Allopurinol (days)								
None	1,016	505	1			1		
<90	125	65	1.09	0.75–1.60	0.644	1.02	0.66–1.56	0.935
90–179	41	24	1.41	0.76–2.64	0.280	1.32	0.67–2.61	0.423
≥180	120	57	0.93	0.63–1.35	0.690	0.89	0.57–1.40	0.623
Benzbromarone (days)								
None	348	181	1			1		
<90	425	219	1.01	0.77–1.32	0.957	0.99	0.73–1.36	0.964
90–179	166	89	1.09	0.75–1.59	0.640	0.90	0.58–1.39	0.643
≥180	363	162	0.74	0.55–0.99	0.045	0.72	0.51–1.02	0.065
Sulfinpyrazone (days)								
None	1,253	626	1			1		
<90	19	9	0.90	0.37–2.21	0.819	1.10	0.41–2.98	0.847
≥90	30	16	1.14	0.56–2.34	0.715	1.49	0.64–3.44	0.353
Probenecid								
None	1,289	644	1			1		
Yes	13	7	1.17	0.39–3.47	0.782	1.34	0.40–4.48	0.631
**Male** Allopurinol (days)								
None	1,312	640	1			1		
<90	258	126	1.00	0.76–1.31	0.996	0.91	0.67–1.25	0.573
90–179	77	44	1.39	0.88–2.21	0.159	1.66	0.96–2.86	0.068
≥180	293	160	1.27	0.98–1.65	0.069	1.14	0.83–1.57	0.404
Benzbromarone (days)								
None	443	245	1			1		
<90	534	268	0.81	0.63–1.04	0.099	0.87	0.65–1.17	0.348
90–179	226	111	0.78	0.57–1.07	0.122	0.80	0.55–1.17	0.252
≥180	737	346	0.71	0.55–0.90	0.005	0.68	0.51–0.91	0.010
Sulfinpyrazone (days)								
None	1,853	923	1			1		
<90	38	21	1.24	0.65–2.34	0.517	1.56	0.74–3.31	0.246
≥90	49	26	1.14	0.64–2.05	0.655	1.36	0.70–2.64	0.364
Probenecid								
None	1,925	959	1			1		
Yes	15	11	2.75	0.88–8.64	0.083	1.75	0.51–6.02	0.372

† *Adjusted for allopurinol, benzbromarone, sulfinpyrazone, probenecid, hypertension, hyperlipidemia, chronic liver disease, chronic kidney disease, diabetes, COPD, autoimmune disease, cardiovascular disease, stroke, depression, parkinson's disease, warfarin, and statin*.

## Discussion

The present study was a large population real-world study investigating the risk of dementia in gout patients using anti-gout preparations. Our findings indicated that use of benzbromarone to treat hyperuricemia and prevent recurrent gout flares in gout patients decreased the risk of incident dementia. Moreover, the protective effect was more pronounced in younger patients.

The ULT for gout patients may not be the same in different countries. In the United States and Europe, gout patients were treated with allopurinol as the preferred first-line agent and are strongly recommended for all patients with normal renal function (26, 27). However, allopurinol can cause skin allergic reactions and fatal exfoliative dermatitis and other hypersensitivity syndromes might develop in severe cases. Human leukocyte antigen (HLA)-B^*^5801 allele positivity, which is significantly higher in Han Chinese than in Caucasians, is a risk factor for the development of adverse reactions to allopurinol, and screening for this gene before initiating allopurinol treatment is important (28, 29). Since Allopurinol is contraindicated in HLA-B^*^5801 allele-positive patients, in Asian areas, allopurinol is usually not the most commonly used ULT (30–32).

Moreover, febuxostat, which can be used in patients with mild-to-moderate CKD, is approved for the treatment of gout in the United States in doses of 40 or 80 mg; by contrast, European approval of febuxostat is for doses of 80 or 120 mg (26, 27). For the choice of uricosuric drugs, probenecid is the only available drug in the United States, but it is neither as efficacious as benzbromarone, which is available in several other countries but not in the United States (26, 33). Benzbromarone, a potent uricosuric drug, was introduced in the 1970s and was registered in about 20 countries throughout Asia, South America, and Europe. In 2003, the drug was withdrawn by Sanofi-Synthélabo, after reports of serious hepatotoxicity and had been not used in American. However, it is still marketed in several countries by other drug companies (33). For example, In Asia (including China and Japan) and Europe, benzbromarone is still the most use uricosuric drugs in gout patients, which is the same as in Taiwan (31, 32, 34). Hence, the use of benzbromarone associated with a reduced risk of future dementia can be a reference in most areas of the world.

In Taiwan, a nationwide population study conducted from 2005 to 2010 reported that the prevalence of gout may be as high as 6.24%, with only 22.93% of patients with gout being prescribed urate-lowering treatment (allopurinol, benzbromarone, probenecid, or sulfinpyrazone) (35). Moreover, among patients who received treatment, 60.08% received uricosuric agents alone (benzbromarone, probenecid, or sulfinpyrazone), 28.54% received xanthine oxidase inhibitor (allopurinol), and 11.38% received combined therapy. In this study, we enrolled gout patients from 2000 to 2008 and is like the epidemiology investigation, most patients use uricosuric drugs than xanthine oxidase (Allopurinol). However, the febuxostat is approved by the U.S. Food and Drug Administration in 2009 and not listed until 2012 in Taiwan. Hence, in the study period, few patients have taken the drug and cannot be enrolled in the analysis.

Risk factors for Alzheimer's disease and vascular dementia are similar to those for CVD (36, 37). Hyperuricemia and gout are also positively associated with CVD (38, 39); therefore, it is rational to postulate with common causality reasoning that gout and hyperuricemia are positively correlated with dementia and that anti-gout preparations have a protective effect. However, previous studies have shown these associations to be controversial.

In 2008, Euser et al. conducted a prospective cohort study investigating the association between serum UA and cognitive function and dementia in patients aged ≥55 years. The data showed higher serum UA levels were associated with a decreased risk of dementia and better cognitive function (40). Previous cohort studies have shown that patients with gout have a lower risk of developing dementia and that this phenomenon exists for both Alzheimer's disease and vascular dementia (41, 42). These studies indicate that the protective effect may be due to the antioxidative effects of UA, which can reduce biological oxidants such as peroxynitrite radicals and protect from neurodegenerative diseases (43). However, this hypothesis remains controversial, as Hershfield et al. did not observe any changes in oxidative stress markers in patients receiving pegloticase, which greatly lowers serum UA levels (44). Furthermore, Desideri et al. reported that UA increased oxidative stress and potentiated the neurotoxic effects of amyloid-β in neuronal cells (45). In contrast, Singh et al. revealed that gout was independently associated with a 15% higher risk of incident dementia (5), and Latourte et al. found that high serum UA levels may increase the risk of incident dementia in the elderly, particularly vascular or mixed type (46). However, while anti-gout preparations are known to lower serum UA levels and prevent gout flare, few studies have focused on the association between anti-gout preparations and dementia.

A recent case-control study showed a slight reduction in the risk of dementia in patients with hyperuricemia, both with and without anti-hyperuricemic treatment (47). In this study, allopurinol was the most frequently prescribed drug (98.4%), followed by benzbromarone (1.8%) and febuxostat (0.2%). Hence, the association between anti-gout preparations and dementia was mostly affected by allopurinol. However, In the present study, the most used drug was benzbromarone, followed by allopurinol. Use of allopurinol was not associated with incident dementia. However, the use of benzbromarone in patients with gout was associated with a lower risk of incident dementia (OR, 0.81).

Benzbromarone is a uricosuric drug that has been widely used since the 1970s as a therapeutic agent for hyperuricemia. Its mechanism of action was identified as suppression of UA resorption via inhibition of urate transporter 1 (18). At standard doses (100 mg/day), the hypouricaemic activity of benzbromarone is more efficacious than that of allopurinol (300 mg/day) and probenecid (1,000 mg/day) and leads to a mean reduction in plasma urate between 25 and 50% (48–51). Moreover, benzbromarone is more effective than probenecid and sulfinpyrazone in lowering UA and can be used in patients with moderate renal insufficiency (52). As expected from any marked reduction in plasma urate concentrations, long-term treatment with benzbromarone is more effective in reducing the frequency of acute gout attacks by 35–100% after 2 years of treatment (53, 54). Besides, patients taking benzbromarone alone achieved a faster reduction of tophi than patients taking allopurinol alone (55). Hence, since hyperuricemia and gout may be risk factors for dementia (5, 46), the more potent effects of benzbromarone in reducing serum UA level and preventing acute gout attacks may explain the protective effect from incident dementia. A recent study by Muraya et al. suggested that benzbromarone had direct antioxidant effects *in vivo* (56), and long-term use of antioxidant supplements can reduce the risk of dementia (57, 58). Hence, this antioxidant effect can also explain why the use of benzbromarone was more effective in reducing incident dementia than other anti-gout preparations.

The present study had some limitations. First, all diagnoses in the NHIRD were made by physicians using ICD-9-CM codes and were mainly registered by general practitioners rather than rheumatologists. Therefore, the definition of gout may have resulted in non-differential misclassification. However, patients were diagnosed with gout following at least two outpatient visits or one admission with the use of anti-gout preparations to ensure that only patients with an accurate diagnosis were selected. Second, information about lifestyles, such as smoking habits and alcohol consumption, was not collected in the insurance database. To reduce this bias, we adjusted for COPD to gauge smoking habits, CLD to reflect alcohol consumption, and other comorbidities. Third, as our data were limited to insurance claims and did not provide further information on treatment and test results of serum UA levels or gout flare, we could not attribute the results to the effect of the drugs. However, previous studies showed that gout patients treated with benzbromarone may have relatively lower serum UA compared with those using other agents.

## Conclusions

The present study investigated patients with gout who were treated with ULT and showed that the use of benzbromarone was associated with a reduced risk of dementia. This effect may be due to the potent UA-lowering and antioxidant effects of benzbromarone. Further prospective trials are warranted to confirm our findings.

## Data Availability Statement

The original contributions presented in the study are included in the article/supplementary materials, further inquiries can be directed to the corresponding author/s.

## Ethics Statement

Ethical review and approval was not required for the study on human participants in accordance with the local legislation and institutional requirements. Written informed consent for participation was not required for this study in accordance with the national legislation and the institutional requirements.

## Author Contributions

All authors listed have made a substantial, direct and intellectual contribution to the work, and approved it for publication.

## Conflict of Interest

The authors declare that the research was conducted in the absence of any commercial or financial relationships that could be construed as a potential conflict of interest.

## Abbreviations

NHIRD: National Health Insurance Research Database
NHI: National Health Insurance
ICD-9-CM: International Statistical Classification of Diseases and Related Health Problems, 9th Revision, Clinical Modification
UA: Uric acid
ULT: Urate-lowering therapy
CLD: Chronic liver disease
CKD: Chronic kidney disease
DM: Diabetes
COPD: Chronic obstructive pulmonary disease
AD: Autoimmune disease
CVD: Cardiovascular disease
PD: Parkinson's disease.
